# Biochemical, Mutational and *In Silico* Structural Evidence for a Functional Dimeric Form of the Ornithine Decarboxylase from *Entamoeba histolytica*

**DOI:** 10.1371/journal.pntd.0001559

**Published:** 2012-02-28

**Authors:** Preeti, Satya Tapas, Pravindra Kumar, Rentala Madhubala, Shailly Tomar

**Affiliations:** 1 Department of Biotechnology, Indian Institute of Technology Roorkee, Roorkee, India; 2 School of Life Sciences, Jawaharlal Nehru University, New Delhi, India; National Institute of Allergy and Infectious Diseases, United States of America

## Abstract

**Background:**

*Entamoeba histolytica* is responsible for causing amoebiasis. Polyamine biosynthesis pathway enzymes are potential drug targets in parasitic protozoan diseases. The first and rate-limiting step of this pathway is catalyzed by ornithine decarboxylase (ODC). ODC enzyme functions as an obligate dimer. However, partially purified ODC from *E. histolytica* (*Eh*ODC) is reported to exist in a pentameric state.

**Methodology and Results:**

In present study, the oligomeric state of *Eh*ODC was re-investigated. The enzyme was over-expressed in *Escherichia coli* and purified. Pure protein was used for determination of secondary structure content using circular dichroism spectroscopy. The percentages of α-helix, β-sheets and random coils in *Eh*ODC were estimated to be 39%, 25% and 36% respectively. Size-exclusion chromatography and mass spectrophotometry analysis revealed that *Eh*ODC enzyme exists in dimeric form. Further, computational model of *Eh*ODC dimer was generated. The homodimer contains two separate active sites at the dimer interface with Lys57 and Cys334 residues of opposite monomers contributing to each active site. Molecular dynamic simulations were performed and the dimeric structure was found to be very stable with RMSD value ∼0.327 nm. To gain insight into the functional role, the interface residues critical for dimerization and active site formation were identified and mutated. Mutation of Lys57Ala or Cys334Ala completely abolished enzyme activity. Interestingly, partial restoration of the enzyme activity was observed when inactive Lys57Ala and Cys334Ala mutants were mixed confirming that the dimer is the active form. Furthermore, Gly361Tyr and Lys157Ala mutations at the dimer interface were found to abolish the enzyme activity and destabilize the dimer.

**Conclusion:**

To our knowledge, this is the first report which demonstrates that *Eh*ODC is functional in the dimeric form. These findings and availability of 3D structure model of *Eh*ODC dimer opens up possibilities for alternate enzyme inhibition strategies by targeting the dimer disruption.

## Author Summary

*E. histolytica* genome sequence divulged the existence of ornithine decarboxylase enzyme that performs the first-rate limiting catalytic step of polyamine biosynthetic pathway. ODC enzyme is a potent therapeutic target in many eukaryotic disease causing pathogens. DFMO, a potent substrate analogue inhibitor, is widely used for the treatment of various diseases including *Trypanosoma brucei* infections. However, DFMO does not inhibit *E. histolytica* ODC. As ODC is a validated drug target for protozoan disease, an alternate strategy to inhibit the *Eh*ODC enzyme may be developed. In our study, we have evidently proved that the purified recombinant *Eh*ODC is functional as an active homodimer. Molecular modeling and simulation studies indicate that two independent active sites are present at the dimer interface. Our mutational studies indicate that the enzyme activity can be abolished by targeting the dimer interface and this in turn suggests the alternative inhibitory mechanism for the enzyme. Our investigation yields that disruption of dimer disrupts the active site pocket and renders the enzyme inactive. As *Eh*ODC crystal structure is unavailable, the 3D structure model of *Eh*ODC homodimer may assist in designing structure based anti-amoebiasis peptides or agents that disrupt the active site by destabilizing the dimer.

## Introduction

Amoebiasis is an infectious disease caused by single-celled parasitic protozoan *Entamoeba histolytica*. Parasitic amoeba infects liver and intestine, which may cause mild diarrhea and serious dysentery with bloody and mucoid stool. If untreated, the parasite can cause life-threatening hemorrhagic colitis and/or extraintestinal abscesses. *E. histolytica* is responsible for over 50 million infections in tropical and temperate regions, and nearly 100,000 deaths worldwide each year [1], [2]. The parasite mainly affects primates and humans, and is transmitted by ingestion of water and food contaminated with feces containing *E. histolytica* cysts. First-line amoebiasis treatment is anti-amoebic therapy that relies on a very small number of drugs such as metronidazole, emetine, tinidazole and chloroquine [3]–[5]. These drugs target different stages of the life cycle of *E. histolytica*. Frequent and widespread usages of these drugs have led to the increase in the minimum inhibitory concentration (MIC) values and also development of clinical drug resistance in pathogen. Some of these drugs have been reported to have significant side effects. For instance, metronidazole, an effective drug for amoebiasis, has been reported to be tumorigenic and mutagenic [6]–[8]. Nitrazoxanide, a broad spectrum anti-parasitic drug used for amoebiasis treatment, is found to be associated with many side effects [9], [10]. Consequently, development of alternate strategies and discovery of new anti-amoebic agents targeting polyamine synthesis is necessary to combat the disease.

**Figure 1:**
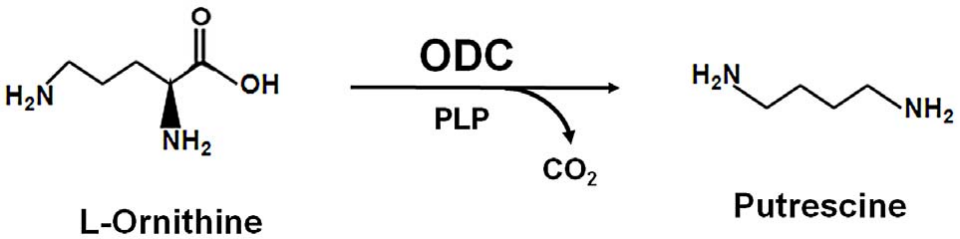
The enzymatic reaction catalyzed by ornithine decarboxylase. The pyridoxal phosphate (PLP)-dependent ODC enzyme catalyzes decarboxylation of ornithine and produces putrescine.

Ornithine decarboxylase (ODC), a Pyridoxal 5′-phosphate (PLP) dependent homodimeric enzyme catalyzes the first rate-limiting step of polyamines biosynthetic pathway by decarboxylation of L-ornithine to form putrescine (Figure 1). Polyamines have an eminent role in various cell growth and differentiation processes [11], [12]. Consequently, ODC being the key enzyme of the polyamine biosynthetic pathway is a promising therapeutic target for anti-protozoan therapy. The ODC enzyme has been reported to be present in various protozoa including *Leishmania*, *Trypanosoma*, *Giardia*, and *Plasmodium* and is a validated drug target in *Trypanosoma brucei* for treatment of African sleeping sickness [13]–[18]. ODC enzyme has a very short half-life due to its ubiquitin-independent 26S proteasome mediated degradation which is stimulated by the binding to antizyme [19]. Besides increase in ODC proteolysis, interaction of antizyme with ODC leads to catalytic inactivation of the enzyme by disrupting the enzymatically active ODC dimers [19], [20]. In addition, the antizyme binding loop which is accessible in ODC monomer is found to be buried in the dimers of ODC that ultimately prevents it from degradation. Thus, dimer formation is not only important for its catalytic function but also for its protection against antizyme-dependent endoproteolysis.

Crystal structures of ODC enzyme from *T. brucei* (PDB ID: 1QU4), human (PDB ID: 2OO0), and mouse (PDB ID: 7ODC) have revealed that the monomeric subunits interact in head to tail manner and form two catalytic active sites at the dimer interface [21]–[23]. The structure of ODC in complex with substrate and product analogues including ornithine analog α-difluoromethylornithine (DFMO) have been investigated [21]. DFMO is a suicide inhibitor of ODC and has been reported to inhibit growth of various pathogenic protozoan parasites such as *Giardia lamblia* [14], *Trichomonas vaginalis* [24], *Plasmodium falciparum*, and various *Trypanosoma* species [13], [18]. In *E. histolytica*, the only enzyme of polyamine biosynthesis reported to exist is ODC. *E. histolytica* ODC (*Eh*ODC) has been reported to form homopentamers [25]. Interestingly, *Eh*ODC is insensitive to DFMO and DFMO has no inhibitory effect on the cell growth of the parasite [25]–[27]. Therefore, it is necessary to develop an alternate method for inhibition of *Eh*ODC enzyme for targeting the polyamine biosynthetic pathway to curb the disease.

In the present work, we have re-investigated the oligomeric state of *Eh*ODC using biochemical, mutational and *in silico* methods. Previously, it has been reported that the *Eh*ODC enzyme exists only as a homopentamer [25]. However, our studies evidently demonstrate that *Eh*ODC is functionally active in the dimeric form. In the absence of crystal structure of *Eh*ODC, we have generated 3D model of *Eh*ODC homodimer to structurally characterize the dimer interface containing two active sites and have performed molecular dynamics simulations to verify the dimer stability. Our investigation yields that disruption of dimer disrupts the active site pocket and renders the enzyme inactive. 3D structure model of *Eh*ODC homodimer may be beneficial in designing structure based anti-amoebiasis peptides or agents that would disrupt enzyme dimerization. We propose that a compound having the capability to disrupt the dimer could be a good candidate for amoebiasis treatment.

## Materials and Methods

### Reagents

The *E. coli* expression vector pET 30a (Novagen) containing full-length gene of *Eh*ODC having N-terminal Histidine tag (6× His) followed by enterokinase cleavage site was used for over-expression of the enzyme [26]. Oligonucleotides for site directed mutagenesis were ordered from Imperial Life Sciences (India). Restriction endonuclease *Dpn*I and Phusion DNA polymerase were acquired from New England BioLab Inc. For protein purification, 5 ml HisTrap HP and HiLoad 16/60 Superdex 200 gel filtration columns were obtained from GE Healthcare. Imidazole (low absorbance at 280) was obtained from Acros. ÄKTA Prime plus system from GE Healthcare was used for protein purification. Putrescine, 4-aminoantipyrine, diamine oxidase (DAO), horseradish peroxidase, L and D-ornithine were procured from Sigma Aldrich. Amicon ultra protein concentrators were purchased from Millipore. All other chemicals were of analytical grade and obtained from commercial sources.

### Over-expression and purification of recombinant *Eh*ODC

The expression and purification of *Eh*ODC enzyme was done by following the published procedure with minor modifications given below [26]. The plasmid pET30a having the full-length *Eh*ODC gene insert (pET30a-*Eh*ODC) was transformed into freshly prepared *E. coli* BL21 (DE3) competent cells and plated on Luria-Bertani (LB) agar plate containing kanamycin (50 µg/ml). Plates were incubated overnight at 37°C and colonies were obtained. Single colony was picked and cells were seeded in 5 ml LB broth containing 50 µg/ml of kanamycin and culture was grown overnight at 37°C with agitation. Overnight culture was used for inoculation of 1 L LB broth. Expression was induced with 1 mM isopropyl β-D-thiogalactoside (IPTG) when optical density (A_600_) reached 0.6. After induction, culture was moved to 18°C and was grown for ∼14 h. Cells were harvested by centrifugation at 5,000 rpm at 4°C for 10 min and cell pellets were stored at −80°C until further processing. Expression and solubility of the protein was confirmed by analysis of lysed cell supernatant and pellet on 12% sodium dodecyl sulfate-polyacrylamide gel electrophoresis (SDS-PAGE).

The histidine-tagged *Eh*ODC was purified using a two step procedure that employed metal ion affinity chromatography followed by gel filtration chromatography. All purification steps were performed at low temperature (4°C–6°C). Briefly, frozen cell pellets from a 1 L culture were thawed on ice and re-suspended in buffer A [50 mM Tris-HCl (pH 7.5), 40 mM imidazole, 250 mM NaCl and 5% glycerol (v/v)] containing lysozyme (0.7 mg/ml) and 0.2 mM phenylmethanesulfonyl fluoride (PMSF). Cells were disrupted by sonication on ice with a pulse of 20 s on and 1 min off for 10 times. The obtained cell lysate was clarified by centrifugation at 18,000 g for 45 min at 6°C and supernatant was applied on HisTrap HP column (5 ml) pre-equilibrated with buffer A. Unbound proteins were removed by washing the column with ∼40 ml of buffer A. Bound protein fractions were eluted using a linear gradient of 40 mM to 1 M imidazole of 60 ml at a flow rate of 1 ml/min. Eluted fractions were examined on 12% SDS-PAGE and fractions containing pure protein were pooled together. To remove the N-terminal His-tag, enterokinase was added to pure protein (∼0.02 units/mg protein) and incubated for ∼12 h at 4°C and simultaneously dialyzed against buffer A without imidazole. To remove uncleaved tagged protein and the cleaved His tags, the sample was reloaded onto HisTrap HP column and the flow-through containing untagged *Eh*ODC was collected and concentrated using a 10 kDa cutoff Amicon Ultra-15 concentrator (Millipore, Bedford, Massachusetts, USA). For removal of enterokinase, the concentrated sample was loaded onto HiLoad 16/60 prep grade Superdex 200 size-exclusion chromatography column pre-equilibrated with buffer B [50 mM Tris-HCl (pH 7.5), 250 mM NaCl, 0.2 mM dithiothreitol (DTT) and 5% glycerol (v/v)]. Fractions of the major peak containing pure protein were pooled and concentrated. Homogeneity of the concentrated enzyme preparation was analyzed by 12% SDS-PAGE. The yield and concentration of purified *Eh*ODC was measured using the Bio-Rad protein-assay kit with bovine serum albumin (BSA) as a standard. *Eh*ODC mutant proteins were expressed and purified using the same protocol.

### *Eh*ODC enzyme assay

Ornithine decarboxylation activity of *Eh*ODC was spectrophotometrically determined by the method developed by Badolo *et al.* [28]. This method is based on the reaction between DAO and putrescine, the product of the ODC-catalyzed reaction. For *Eh*ODC enzyme assay, the purified protein was buffer exchanged with 20 mM sodium phosphate buffer (pH 7.5) and concentrated to final concentration of 0.3 mg/ml. The reaction mixture of 180 µl containing 20 mM sodium phosphate buffer (pH 7.5), 0.1 mM EDTA, 0.1 mM PLP, 0.2 mM DTT, and 1 mM of L-ornithine was prepared to which 20 µl of protein solution was added to make up the final volume of 200 µl. The reaction mixture was incubated at 37°C for 5 h. Further, 100 µl of the above *Eh*ODC reaction mixture was added to 900 µl of diamine oxidase (DAO) reaction mixture composed of 50 mM Tris-HCl (pH 9.8) containing 100 µg/ml phenol, 100 µg/ml 4-aminoantipyrine (4-AAP), 0.02 U of DAO, and 7 U of horseradish peroxidase (HRP). The reaction was incubated at 25°C for 60 min and then terminated by heating the solution at 90°C for 4 min. The concentration of putrescine formed by ornithine decarboxylation catalysis was determined by measuring the absorbance at 492 nm for the colored complex formed as a result of the reaction of H_2_O_2_ with 4-AAP and phenol catalyzed by HRP. For negative controls, purified protein or substrate L-ornithine were substituted with buffer in the ODC enzyme reaction mixtures. Effect of stereoisomer of substrate was observed by incubation of L and D-ornithine at 37°C.

### Glutaraldehyde crosslinking

To obtain preliminary information on the oligomeric association of *Eh*ODC, glutaraldehyde crosslinking experiment was performed using the method described by Fadouloglou *et al.* [29]. Purified protein solution was exchanged with 20 mM sodium phosphate buffer (pH 7.5) for cross-linking studies. Experiment was carried out using 24 well crystallization plate (Hampton research) and a siliconized coverslip in a manner similar to a hanging drop crystallization method. For cross-linking *Eh*ODC, 40 µl of 12.5% glutaraldehyde solution (v/v) acidified with 1 µl 5 N HCl was added in the well of crystallization plate. Then, 15 µl of protein solution (1 mg/ml) was loaded onto the coverslip, which was inverted on the reservoir well and sealed with vacuum grease (Hampton Research). The entire setup was incubated at 37°C for 10 min and then the sample was mixed with an equal volume of 2X SDS-PAGE loading buffer and boiled for 4 min on a dry bath. Cross-linked oligomers were analyzed on 12% SDS-PAGE followed by Coomassie Blue R-250 staining.

### Molecular mass and oligomeric state determination

The molecular mass of recombinant *Eh*ODC was determined by running purified protein on 12% SDS-PAGE with standard molecular weight protein marker (Bio-Rad). To analyze the oligomerization state, 500 µl of purified and concentrated (∼10 mg/ml) protein was applied onto a HiLoad 16/60 Superdex 200 gel filtration column pre-equilibrated with buffer B using 500 µl sample loop at a flow rate of 0.5 ml/min on ÄKTA purifier chromatographic system (GE Healthcare) and protein elution profile was monitored by measuring the absorbance at 280 nm. The size-exclusion column was calibrated with blue dextran (2000 kDa), and Gel Filtration HMW Calibration kit containing ferritin (440 kDa), aldolase (158 kDa), Conalbumin (75 kDa) and ovalbumin (43 kDa) (GE Healthcare) for determination of the void volume, construction of the standard curve and estimation of the molecular weight of purified protein.

The oligomerization state of *Eh*ODC was also analyzed by matrix-assisted laser desorption/ionization time of flight mass spectrometry (MALDI/TOF MS). The purified protein sample was dialyzed against 50 mM Tris buffer (pH 7.5) containing low concentration of NaCl (25 mM) and 0.2 mM DTT to avoid any instrumental interference and was concentrated to ∼2 mg/ml using 10 kDa cutoff Amicon ultra 15 (Millipore). The MALDI/TOF MS analysis was carried out at Proteomics Facility, TCGA (New Delhi, India) using Ultraflex mass spectrometer (Bruker Daltonics, Germany). The protein ionization spectra were analyzed on FLEX-PC2 mass spectrometer and data was acquired across the range of about 0 to 250 amu.

### Effect of Urea and NaCl on *Eh*ODC oligomerization

To study the effect of urea and NaCl on oligomeric state of protein, purified and concentrated *Eh*ODC was pre-incubated with variable concentration (2 M or 4 M) of above chemical agents separately at 4°C for 4 h. The protein was further loaded onto Hi-load 16/60 superdex 200 gel filtration column equilibrated with Buffer B containing the same concentration of urea or NaCl and elution profiles were analyzed.

### Far-UV Circular Dichroism spectrum

For estimation of secondary structure elements, purified *Eh*ODC was subjected to circular dichroism (CD) analysis using Chirascan Circular Dichroism Spectrometer (Applied Photophysics Ltd., Surrey KT22 7PB, United Kingdom). CD spectra were collected using a 1 mm quartz cell under constant nitrogen purge between 190 to 260 nm in 0.5 nm wavelength steps and an average time of 3.0 s at 25°C. The protein solution was buffer exchanged with 20 mM potassium phosphate buffer (pH 7.5) at 4°C. Protein samples at concentration 0.35 mg/ml were analyzed and three scans were collected, averaged and the baseline corresponding to the above buffer was subtracted to obtain the final values. The obtained data were analyzed using the software K2d (http://www.embl.de/~andrade/k2d.html) [30].

### Site directed mutagenesis

The pET30a-*Eh*ODC plasmid containing *Eh*ODC gene was mutated using the QuikChange XL mutagenesis kit by following the instructions of manufacturer (Stratagene, La Jolla, CA). Mutations were introduced into the synthetic mutagenic oligonuclotide primers and were used for construction of mutant plasmids. Mutations and respective mutagenic primers are listed in table (Table 1). The pET30a-*Eh*ODC plasmid was used as a template in the primer extension reaction for constructing the mutants. The reaction mixture used for PCR amplification contained 10 µl of 5X HF phusion buffer supplied with the enzyme, 300 µM of dNTP mix, 6.25 pmol of each primer, 10 ng of template DNA, 2.5 U of phusion polymerase, and water was added to make up the final volume of 50 µl. PCR reaction was performed by subjecting the samples to 20 cycles of 30 s denaturation at 95°C, 1 min at annealing temperature as given in Table 1, and 6 min 50 s elongation at 72°C, and finally reaction was completed by doing extension for 15 min at 72°C. PCR products were analyzed on 1% agarose gel electrophoresis. The parent methylated template plasmids were digested with *Dpn*I restriction enzyme at 37°C for 1 h. Digested product was directly used to transform XL-1 Blue competent cells. Transformed cells were plated on LB agar plate containing 50 µg/ml of kanamycin and plates were incubated at 37°C for ∼16 h. The presence of the mutations in the constructed plasmids were confirmed by DNA sequencing using T7 promoter or terminator universal primers at genomic and proteomic facility of TCGA (New Delhi, India).

**Table 1:** Sequence of mutagenic primers and annealing temperature used for PCR amplification of mutant plasmids.

Mutants	Nucleotide sequence	Annealing Tm
Lys57Ala (S)	CTTGCTTTGCTGTTGCATGTAATCCTGAACCTCA	53°C
Lys57Ala (An)	TGAGGTTCAGGATTACATGCAACAGCAAAGCAAG	
Cys334Ala (S)	GTATTATTTATGGACCTTCTGCTAATGGAAGTGATAAAG	57°C
Cys334Ala (An)	CTTTATCACTTCCATTAGCAGAAGGTCCATAAATAATAC	
Gly361 Tyr(S)	GGTTATTATTTCCCAATATGTATGCTTATACAATTTC	50°C
Gly361Tyr (An)	GAAATTGTATAAGCATACATATTGGGAAATAATAACC	
Lys157Ala (S)	ATGTATTTGGAGAGGCATTTGGACTTCATGATGA	58°C
Lys157Ala (An)	TCATCATGAAGTCCAAATGCCTCTCCAAATACAT	

Mutated nucleotides are underlined. S: sense and An: antisense.

### Phylogenetic and sequence analysis

The ODC sequence of *E. histolytica* was retrieved from NCBI database. Blast and PSI-blast search were performed using AAX35675.1 as query against the non redundant protein sequence database to identify and analyze orthologous sequences. These homologous sequences were retrieved from the NCBI database and multiple sequence alignment was generated using ClustalW and compared for phylogenetic analysis [31].

### Molecular modeling

Three-dimensional (3D) homology model of *Eh*ODC homodimer was generated by comparative modeling using MODELLER 9v8 [32]. To obtain an effectual model, five sequential steps were performed: template selection from Protein Data Bank (PDB), sequence-template alignment, model building, refinement of the obtained model and validation. Template search was done using NCBI BLAST search tool for PDB database [33]. BLASTP algorithm was run with BLOSUM62 as a scoring matrix. Crystal structure of human ODC (PDB ID: 2OO0) which has 34% sequence identity with *Eh*ODC was selected as a template for structure modeling [23]. The graphically enhanced alignment with secondary structures were obtained using ESPript 2.2 server [34].

MULTALIN server was used to align the query sequence with the template sequence [35]. Some manual corrections were done in the alignment file for missing residues in the template sequence. The cofactor, PLP was incorporated into the modeled structure of *Eh*ODC from the template structure and five preliminary models were generated using MODELLER 9v8. All models were selected on the basis of lowest DOPE scores and assessed sterio-chemically by PROCHECK [36]. Energy minimizations of the best chosen models were performed using Swiss-PDB Viewer4.01 (http://www.expasy.org/spdbv/). Loop refinement module of the MODELLER was used for the refinement of the disorganized residues in loops and refinement process was considered for structure validation. Each refined model was verified using ERRAT plot which gives the measure of structural errors in each model at residue level in the protein (http://nihserver.mbi.ucla.edu/SAVES/). The refined model was further validated by ProSA energy plot and VERIFY-3D of the SAVES server [36], [37]. All the graphical visualization and image production were performed using PyMOL [38].

### Molecular dynamics simulation

Molecular dynamics (MD) simulation of dimeric model of *Eh*ODC was performed using GROMACS (v 4.5.4) package [39]. GROMOS96 43a1 force field and 47324 SPC water molecules for solvation of protein were used for simulation. The molecule was solvated in a cubic box at a distance of 1.0 nm between the proteins and the box edge. Electrostatic interactions were calculated using the Particle-mesh Ewald method. Van der Waal and coulomb interactions were truncated at 1 nm. Molecule was neutralized by adding 24 Na^+^ counter ions to the surface and was allowed to undergo 1000 energy minimization steps. All bond lengths including hydrogen atoms were constrained by the LINCS algorithm. To maintain the system at isothermal and isobaric conditions of 300 K and 1 bar, a V- rescale and Parrinello-Rahman barostat coupling was applied for 100 ps. Following to the equilibration, MD simulation was initiated for 1 ns and then extended to 8 ns, with all trajectories sampled at every 1.0 ps.

## Results and Discussion

### Sequence analysis and phylogeny

**Figure 2:**
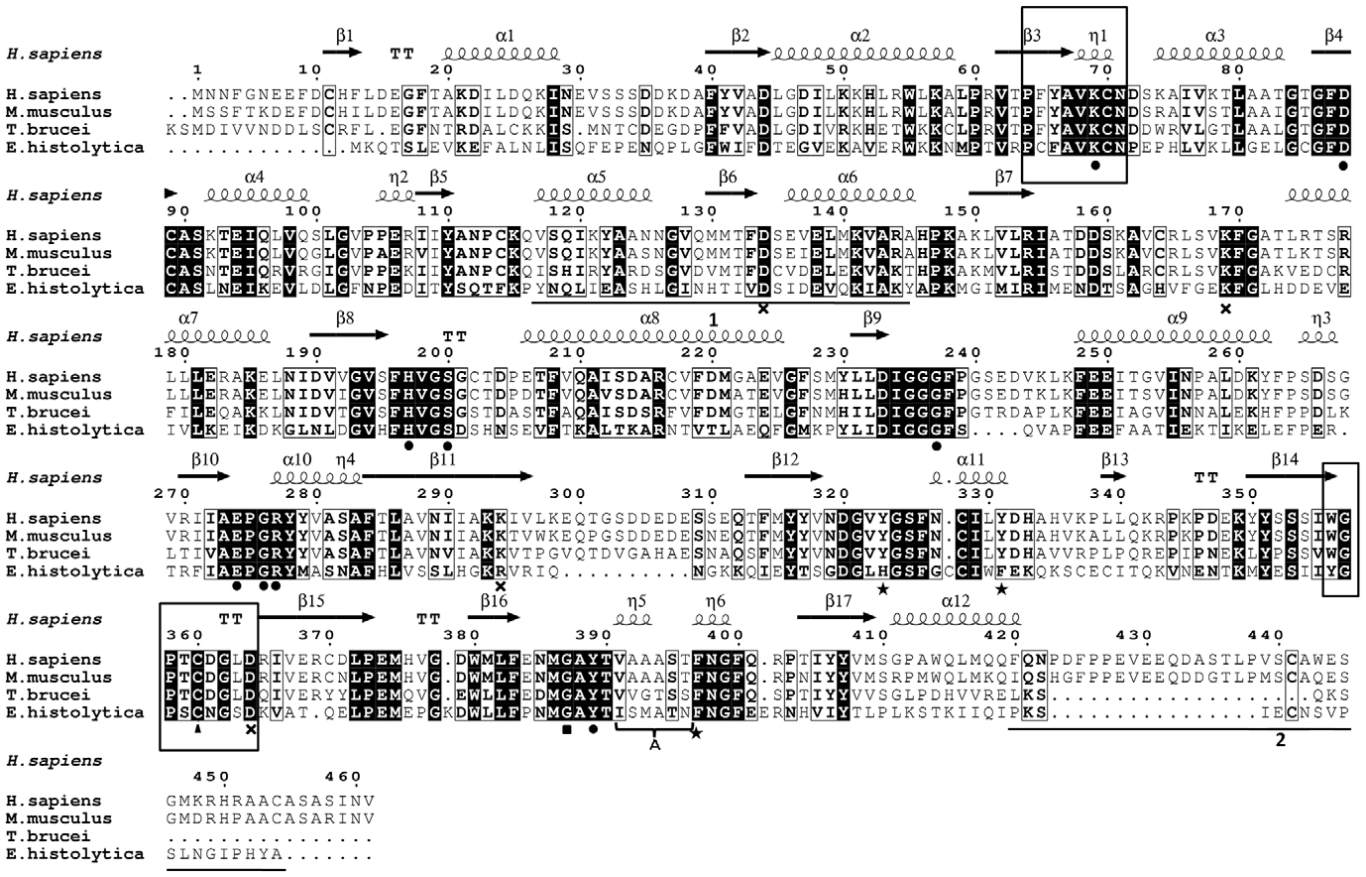
Multiple sequence alignment of *Eh*ODC (AAX35675) with other ODC sequences. The conserved residues are highlighted with black background color. The secondary structure elements and numbering of amino acid sequence of human ODC are presented above the aligned sequences. The signatory motifs PxxAVKC(N) (PLP binding motif) and WGPTCDGL(I)D (substrate binding motif) are highlighted in boxes where “x” signifies any amino acid and amino acids in brackets depict the option at a given position. Underlined sequence denotes the amino acids showing similarity with (1) Antizyme binding region (2) PEST like region. The circles under the amino acid indicate the residues interacting with cofactor PLP where as triangles denote the substrate L-ornithine binding residues in the active site pocket. The residues denoted with cross mark are involved in formation of salt bridges in between two monomers. The residues indicated with stars are present at the interface and form a stack of aromatic rings. Residue important for dimer formation and present away from the interface is denoted with a square. The motif A represents the interface residues of two monomers present very closer to each other. Alignments are obtained using ESPript.

**Figure 3:**
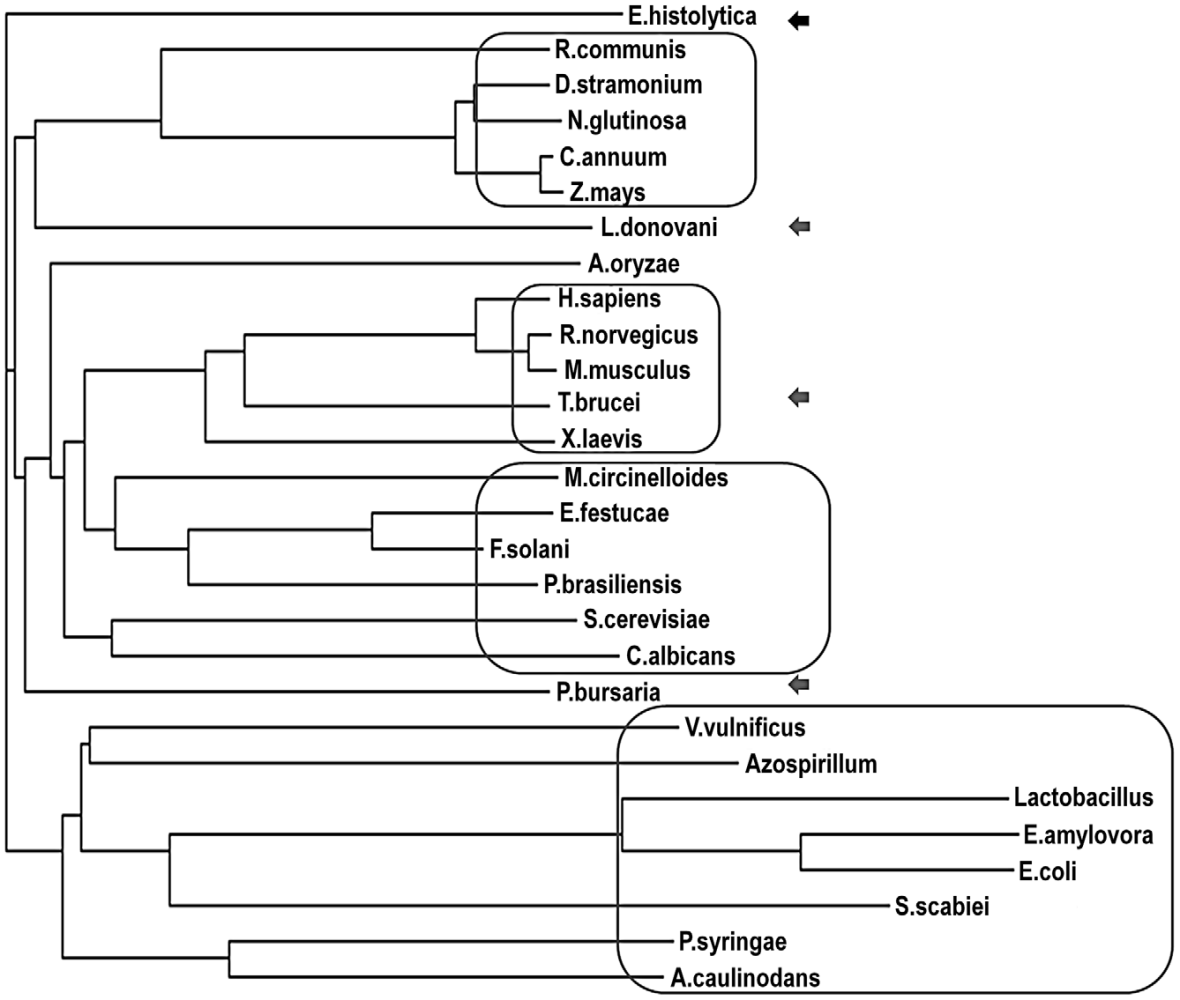
Phylogeny of ornithine decarboxylase from various sources. The amino acid sequences of ODC were taken from plants *R. communis* (XP_002510610.1), *N. glutinosa* (AAG45222.1), *C. annum* (AAL83709.1), *Z. mays* (AAM92262.1), *D. stramonium* (P50134.1); animals *X. laevis* (NP_001079692.1), *R. norvegicus* (NP_036747.1), *M. musculus* (P00860.2), *H. sapiens* (P11926.2); fungi *A. oryzae* (XP_001825149.2) *M. circinelloides* (CAB61758.1), *E. festucae* (ABM55741.1), *P. brasiliensis* (AAF34583.1), *S. cerevisiae* (EDN60096.1) *F. solani* (ABC47117.1), *C. albicans* (AAC49877.1); protozoa *P. bursaria* (NP_048554.1), *T. brucei* (P07805.2), *L. donovani* (P27116.1), *E. histolytica* (AAX35675) and bacteria *V. vulnificus* (YP_004188159.1), *A. caulinodans* (YP_001523249.1), *P. syringae* (AAO58018.1), *E. amylovora* (YP_003539917.1), *S. scabiei* (YP_003491041.1), *Azospirillum* (BAI72082.1), *E. coli* (BAE77028.1), *Lactobacillus* (P43099.2). Different clusters representing a particular group are highlighted in boxes where as the representatives of protozoa ODC are highlighted by arrow marks.

The completion of genome sequence project of *E. histolytica* headed by the Institute of Genome Research (TIGR, Rockville, USA.) opened up the possibilities of new therapeutic targets as well as detailed mechanisms of various biosynthetic pathways [40]. The polyamine biosynthesis in *E. histolytica* is an essential pathway required for the existence of the pathogen [11], [12]. In present study, the sequence of *Eh*ODC, the first and rate-limiting enzyme of polyamine biosynthetic pathway, has been retrieved from NCBI database with accession number AAX35675. The protein consists of 413 amino acids with predicted molecular weight of 46.43 kDa. In *E. histolytica*, the gene encoding ODC is of 1242 bp, thus it implies that there is no intron present in the gene. The enzyme has been previously characterized by Jhingran *et al*. [26]. The amino acid sequence alignment of *Eh*ODC with representative ODCs from different sources revealed that the active site residues along with dimer interface residues responsible for dimerization are highly conserved (Figure 2). *Eh*ODC showed overall 36 to 39% identity with plants, 15 to 25% with bacteria, 35 to 38% with fungi and 32 to 38% with animals. Interestingly, *E. histolytica*, being a protozoan was expected to show high sequence identity, but surprisingly it shows same range of identity with other protozoa including *T. brucei*, *Dictyostelium dasciculates* and *P. falciparum*, etc. i.e. 32 to 35%. From phylogenetic tree, the ODC from plants, fungi, and bacteria make different clusters on the basis of sequence homology where as the protozoan ODCs do not cluster together, instead are distributed throughout showing resemblance with bacteria, fungi and plants (Figure 3). However, *Eh*ODC shows maximum homology with plant ODCs and the evolutionary origin of *Eh*ODC or protozoan ODCs on the basis of phylogenetic analysis is not conclusive. Nevertheless, sequence analysis shows conservation of dimer interface residues which specify the possibility of *Eh*ODC enzyme dimerization similar to other ODCs.

Further sequence analysis revealed that the substrate binding motif having a consensus sequence WGPTCDGL(I)D is highly conserved in human, mouse and *T. brucei* and Cys plays a critical role in catalysis. However, in *Eh*ODC, though Cys is conserved, but the sequence exists as 330 YGPSCNGSD 338 (Figure 2).

The regulation of ODC activity is partially modulated by antizyme-induced, ubiquitin-independent degradation by the 26S proteasome, mainly found in mammals [20], [41]–[43]. Antizyme binds to the inactive ODC monomer forming a hetero-dimer complex which promotes proteolysis degradation [20], [44]. In human ODC, the antizyme binding locus consists of 30 residues at N-terminal ranging from 115Lys to 144Arg residues. The same locus is also highly conserved in mouse. However, this locus in *Eh*ODC which corresponds to 105Tyr to 132Lys having 23% identity is not conserved. In this locus, three residues 121Lys, 141Lys and 144Arg (in human ODC) are highly conserved and responsible for antizyme binding [22]. However, in *Eh*ODC, 121Lys and 144Arg are substituted by 109Ile and 132Lys respectively. Thus, it may be possible that these differences in sequence makes *Eh*ODC insensitive or poorly sensitive to antizyme binding as antizyme dependent ODC degradation has not been reported in *E. histolytica* till date.

Addition to this, in mouse ODC two basal degradation elements (376 to 424 and 422 to 461) at C-terminal are reported which are rich in proline (P), glutamic acid (E), serine (S), and therionine (T) called PEST sequence [23]. In this region, C441 (in both mouse and human ODC) is identified to be a critical residue that promotes polyamine-dependent proteolysis [20], [45]. Similar pattern of sequence arrangement is also observed in *Eh*ODC where it ranges from 395 to 413, and conserved Cys400 corresponds to Cys441 in mouse ODC.

### *Eh*ODC purification and enzyme activity

**Figure 4:**
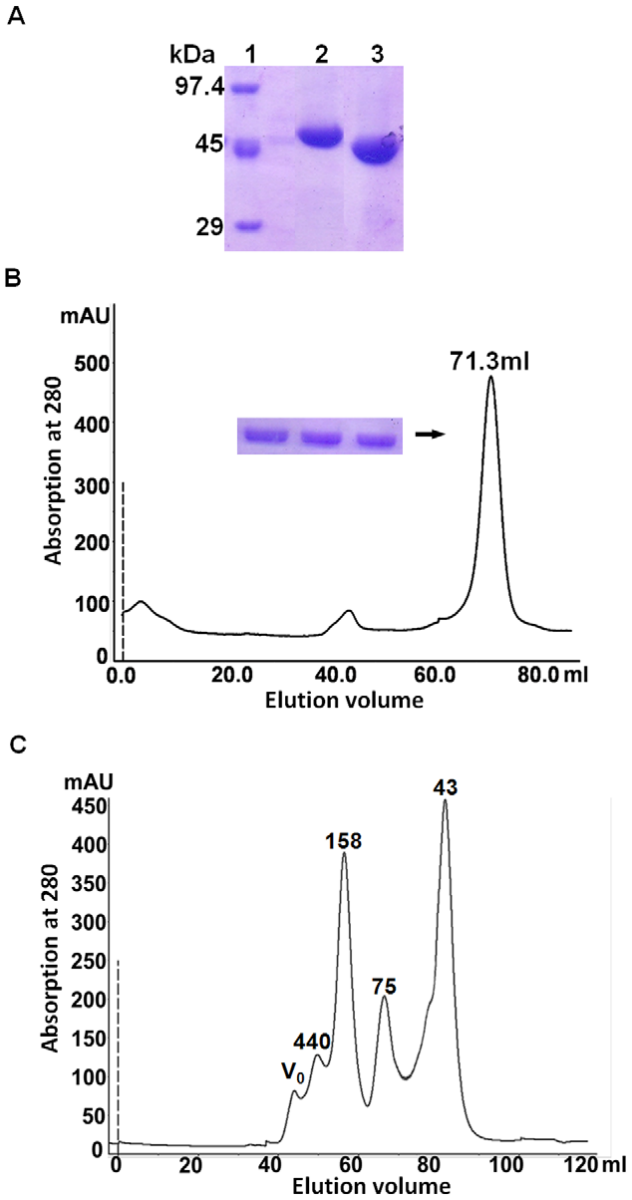
Purification and molecular mass determination of *Eh*ODC. (A) Affinity purification of *Eh*ODC showing purified protein in 12% SDS-PAGE. Lane 1: Molecular weight marker; Lane 2: Purified *Eh*ODC-His tagged protein; Lane 3: Purified His tag cleaved protein with molecular weight ∼46 kDa. (B) Size-exclusion chromatography profile of *Eh*ODC and 12% SDS-PAGE (insert) analysis of major peak fractions. (C) The elution profile of standard molecular weight markers from size exclusion chromatography through HiLoad 16/60 Superdex 200 column. The column void volume (V_o_) and molecular weight (kDa) of standard proteins are indicated.

The recombinant *Eh*ODC protein was purified to homogeneity using two step procedure consisting Ni^2+^ affinity chromatography and size exclusion chromatography. The crude containing over-expressed *Eh*ODC from *E. coli* having N-terminal His-tag was loaded onto HisTrap Ni^2+^ column and eluted using a linear gradient of imidazole. The N-terminal His-tag from eluted protein sample was removed using enterokinase and sample was re-loaded onto HisTrap Ni^2+^ column. Then, the flow-through containing *Eh*ODC without His-tag was collected, concentrated and loaded onto HiLoad 16/60 superdex 200 gel-filtration column for further purification. Homogeneity of pure protein sample was estimated on 12% SDS-PAGE, which exhibited a single band of ∼46 kDa corresponding to the molecular weight of *Eh*ODC protein (Figure 4). The yield of the purified protein was estimated to be ∼3.0 mg/L of culture and protein was concentrated to ∼6 mg/ml.

The enzymatic activity of purified protein was demonstrated using the simple and rapid colorimetric ODC activity assay [28]. The decarboxylation activity of purified enzyme was assayed in 200 µl reaction containing 20 mM sodium phosphate buffer (pH 7.5), 0.1 mM EDTA, 0.1 mM PLP and 1 mM of L-ornithine. The reaction was assayed in terms of the formation of product, putrescine by its oxidation using DAO enzyme which releases H_2_O_2_ that forms a colored complex as described in materials and methods. His-tagged and untagged protein showed no difference in the enzymatic activity. Furthermore, the purified *Eh*ODC actively catalyzed the conversion of L-ornithine to putrescine, while it showed no activity when D-ornithine was used as a substrate in enzyme reaction. This reveals that *Eh*ODC enzyme is stereospecific in binding to L-ornithine substrate suggesting that substrate based stereospecific inhibitors may be designed for *Eh*ODC.

### Secondary structure analysis of *Eh*ODC

**Figure 5:**
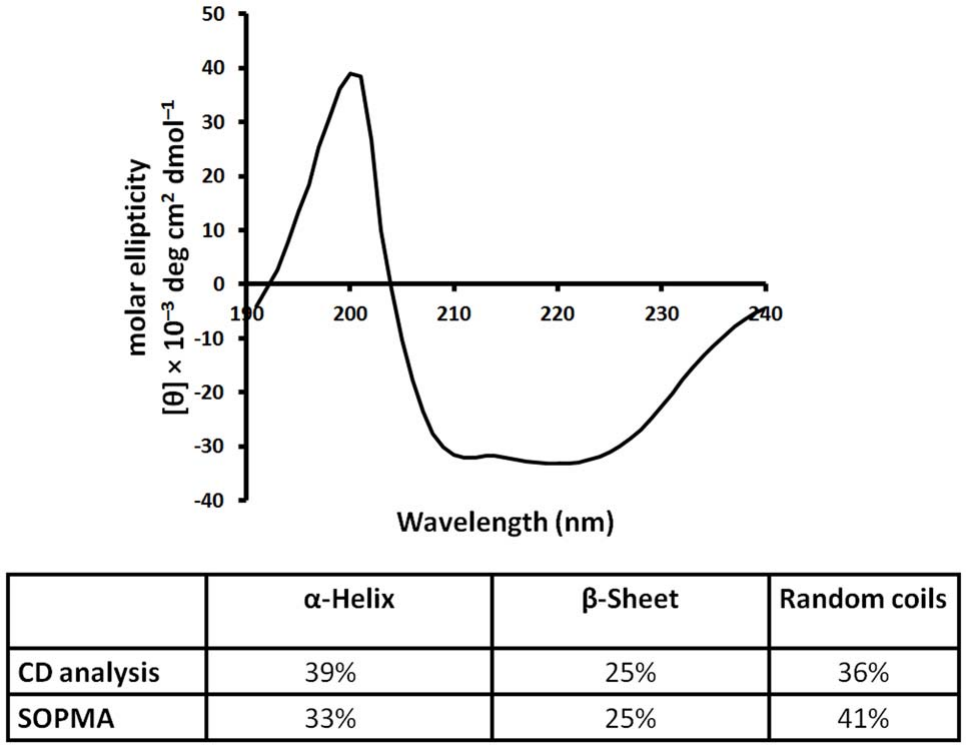
Circular Dichroism spectroscopy of *Eh*ODC. A Far-UV CD spectrum of 0.35 mg/ml *Eh*ODC. Data was analyzed using online K2d server for determining the secondary structure contents. Inserted table shows the comparative secondary structure content obtained by CD data analysis and SOPMA server.

An effort was made to elucidate the secondary structure of *Eh*ODC by using Far-UV circular dichroism (CD). CD spectrum analysis of *Eh*ODC exhibits two negative peaks at 211 and 219 nm and a positive peak in the range of 192-203 nm, as expected for a protein with α/β content, indicating that purified protein has a well defined structure (Figure 5). The deconvolution of CD data with K2d program indicates a secondary structural content of 39% α-helix, 25% β-sheet, and 36% random coil (http://www.embl.de/~andrade/k2d.html) [30]. For comparative secondary structure analysis, the server SOPMA was used for the prediction of secondary structural elements in *Eh*ODC sequence [46]. K2d results were found to be in agreement with the result of SOPMA showing 33% α-helix and 25% β-sheet content (Figure 5). These estimations are in accordance with the available crystal structures of ODCs and also with the molecular model for *Eh*ODC, which was generated by homology modeling in the present study. These results reveal that *Eh*ODC contains an α/β tertiary structure and has the overall folding pattern similar to the other ODCs from mammals, plants and protozoa.

### Characterization of oligomeric state of wild type *Eh*ODC

ODC purified from *E. histolytica* has previously been reported to exist in a pentameric state [25]. Three dimensional crystal structure studies of ODCs from different sources have shown that the enzyme exists as a homodimer and association of monomeric subunits directs the formation of two equivalent catalytic pockets at the dimer interface. Structural analysis revealed that each active site at the dimer interface is assembled by amino acid residues contributed from each monomer subunit, which has also been confirmed by mutational studies [21]–[23]. Therefore, we were interested in characterizing the functional oligomeric form of *Eh*ODC. To accomplish this, we purified recombinant *Eh*ODC enzyme and first confirmed that the purified protein is enzymatically active.

**Figure 6:**
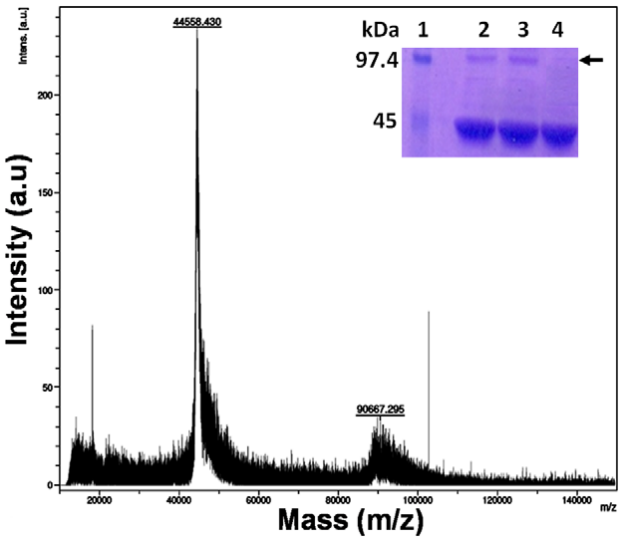
Oligomeric state determination. MALDI-TOF MS analysis of *Eh*ODC showing two peaks corresponding to ∼44558.430 Da and ∼90667.295 Da. The insert shows 12% SDS-PAGE analysis of glutaraldehyde crosslinked *Eh*ODC. Lane 1: Molecular weight markers; Lane 2–3: Protein treated with glutaraldehyde and the two bands correspond to dimer (∼90 kDa) and monomer (∼46 kDa). Arrow points to the crosslinked dimer of *Eh*ODC; Lane 4: Purified protein not treated with glutaraldehyde.

Cross-linking agent, glutaraldehyde is used for obtaining crude information about the quaternary structure of proteins [29]. Previously, the crosslinking experiment has been performed to reveal the dimeric form of mouse ODC [47], [48]. Therefore, *Eh*ODC was cross-linked using glutaraldehyde in a closed setup similar to protein hanging drop crystallization method. After incubation for 10 min, the protein sample was analyzed using SDS-PAGE. The cross-linked sample showed two bands of ∼90 kDa and ∼46 kDa corresponding to the molecular weight of *Eh*ODC dimer and monomer (Figure 6) indicating the possibility of *Eh*ODC dimerization.

To further analyze *Eh*ODC oligomerization, the molecular weight of purified protein was estimated by applying the sample onto a HiLoad 16/60 prep grade Superdex 200 gel-filtration column using ÄKTA purifier. Purified protein showed a major peak with the elution volume 71.3 ml (Figure 4). Using a standard curve based on molecular weight markers, the molecular weight of major elution peak was calculated and was estimated to be approximately ∼90 kDa, which corresponds to the molecular weight of *Eh*ODC dimer (Figure 4). This suggests that *Eh*ODC exists in the dimeric form. Furthermore, MALDI/TOF MS analysis of the purified protein was carried out to verify and confirm the dimerization of protein. MS data showed two narrow peaks having average intensity of 44558.430 m/z and 90667.295 m/z and these correspond to the monomeric and dimeric state of the protein respectively (Figure 6). Thus, it was established that *Eh*ODC enzyme exists in dimeric state.

**Figure 7:**
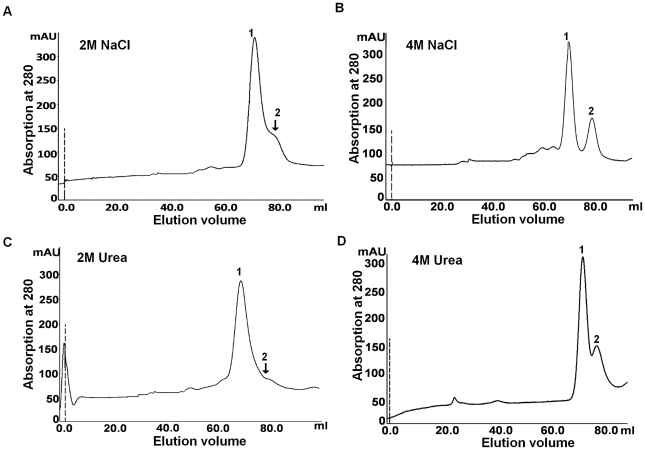
Effect of chaotropic agents on oligomeric property of *Eh*ODC. (A) & (B) Gel-filtration chromatogram showing the elution profile of *Eh*ODC protein treated with 2 M and 4 M NaCl respectively; (C) & (D) Gel filtration chromatogram showing the profile of protein treated with 2 M and 4 M urea respectively.

The study of effect of chaotropic agents on oligomeric state is critical to evaluate the stability of quaternary structure of proteins. The behaviour of ODC in presence of such agents differs from species to species and dissociation of oligomeric state is dependent on the concentration of chaotropic agents [49], [50]. In *T. brucei*, ODC dissociates into monomers in presence of high concentration of salt and urea [51]. This provoked us to examine the effect of different concentrations of NaCl and urea on oligomeric state of *Eh*ODC. Incubation of protein sample with 2 M and 4 M of NaCl resulted in partial dissociation of dimeric enzyme to monomeric state (Figure 7). Two peaks were observed in gel filtration chromatogram: one at 71 ml elution volume followed by a smaller peak at 81 ml elution volume which correspond to the molecular mass of the dimeric and monomeric forms of *Eh*ODC respectively (Figure 7). With increased concentration of NaCl from 2 M to 4 M, the small peak corresponding to monomer becomes more distinct demonstrating that higher concentration of NaCl partially disrupts the dimerization. This also suggests the role of inter-molecular salt-bridges and weak polar interactions in *Eh*ODC dimerization. Similar results were observed when the protein was treated with 2 M and 4 M urea (Figure 7). Destabilization of *Eh*ODC dimers in higher urea concentration points to the presence of inter-molecular hydrophobic interactions at the dimer interface.

### Generation and stability of 3D molecular model of *Eh*ODC

The molecular structure and subunit interactions in *Eh*ODC were investigated by constructing a dimeric model of the enzyme using homology modeling approach. The sequence homology search for *Eh*ODC gave the hits of 29 sequences against PDB database. The crystal structure of human ODC was the first hit with 34% sequence identity (PDB ID: 2OO0) followed by *Tb*ODC (33%, PDB ID 1QU4). For comparative homology modeling, it could be significant to select a template for ODC from protozoan source i.e. *Tb*ODC. However, too much variations in the sequences of ODC within protozoa (Figure 2) and higher sequence identity of *Eh*ODC with plant and mammalian ODC, give an indication of caution required in the interpretation of template selection. Here, we have selected human ODC as template for a reliable model generation considering two major facts: firstly, the N-terminal loop region consisting of approximately eight amino acids is missing in all crystal structures of ODC except human ODC. Secondly, multiple sequence alignment analysis showed a PEST like sequence in the C-terminal region of *Eh*ODC sequence that has maximum similarity with human ODC (Figure 2). The model for *Eh*ODC along with its cofactor PLP was generated from PDB 2OO0 as a template using Modeller 9v8 and model with lowest DOPE score was considered for further loop refinement using Modeller loop refinement tool. The model was subjected to energy minimization where PROCHECK, ERRAT plot and ProSA energy plot were used for validation and quality assessment of the model. The root-mean-square deviation (RMSD) of Cα atoms between the modeled *Eh*ODC dimeric structure and the template structure was 0.744Å. Ramachandran plot of the model generated by PROCHECK showed 90.3% residues in the core region, 7.8% in allowed region, 0.6% in generously allowed region and 0.3% in disallowed region. The generated models have been submitted to Protein Model database (PMDB) with PMDB id: PM0077698 (monomer) and PM0077699 (dimer).

The molecular model of *Eh*ODC dimer that was generated using the crystal structure of human ODC dimer as a template was MD simulated for 8 ns in equilibration with water molecules. Evaluation of the dimer stability was made by monitoring the root-mean-square deviations (RMSD) of the Cα of the dimer which was computed against the starting structure. Analysis of MD trajectory of *Eh*ODC homodimer revealed that RMSD value increases to 0.327 nm in about 1.2 ns and this plateau value is stable till the end of the simulation indicating a stable conformation of the dimer (data not shown).

### Structure analysis of *Eh*ODC monomeric subunit

**Figure 8:**
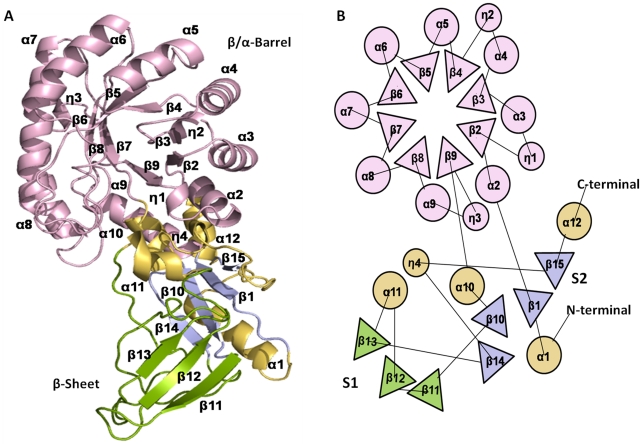
3D structure of *Eh*ODC monomer. (A) Cartoon diagram of *Eh*ODC model generated using Modeller 9v8. (B) Topological arrangement of secondary structures in *Eh*ODC monomer. Monomer of *Eh*ODC consists of two domains, β/α-barrel shown in purple and sheet domain having sheet S1 in green, sheet S2 in blue and helices and turns in orange. The helices are presented by circles, strands are represented by triangles and the loops connecting these structures are represented as connecting lines.

Structure of *Eh*ODC monomer subunit is comprised of two major domains i.e. β/α-barrel and β-sheet domain which is a distinct characteristic of ODC structure (Figure 8). In human ODC, N-terminal starts with a β-strand while in *Eh*ODC, it starts with α-helix. The N-terminal emerges from β-sheet domain and enters the barrel through a coil connecting both the domains. The barrel contains eight parallel strands each followed by a helix in the order α_2_β_2_, η_1_α_3_β_3_, α_4_η_2_β_4_, α_5_β_5_, α_6_β_6_ α_7_β_7_, α_8_β_8_ and α_9_η_3_β_9_. One important feature observed in *Eh*ODC is the presence of turns in a pattern at the N-terminal barrel secondary structures. Such pattern has been observed in ODC like antizyme inhibitor proteins that have structures similar to ODC, but do not possess decarboxylation activity [52]. The sheet domain is subdivided into two clusters of sheets S1 and S2 as observed in all ODC structures. These sheets S1 and S2 remain perpendicular to each other having four helices with one turn (α_1_, α_10_, α_11_, α_12_ and η_4_) around it. Sheet S1 includes three parallel β-strands (↓β_11_, ↑β_12_ and ↑β_13_) which extends into S2 containing four parallel β-strands (↓β_10_, ↑β_14_, ↑β_15_ and ↑β_1_) (Figure 8).

### Structure analysis of dimeric *Eh*ODC

**Figure 9:**
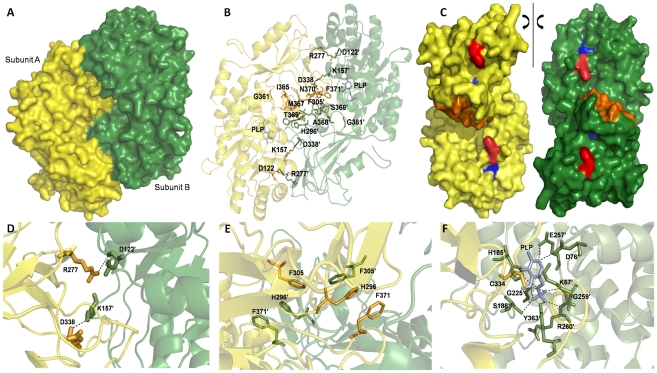
Schematic representation of dimer interface and active site of *Eh*ODC. (A) Subunits of the dimer are arranged in head to tail manner where subunit A and B are shown in yellow and green colors respectively. (B) The residues critically important for dimer formation are presented in sticks and overall dimeric structure is presented in cartoon. Residues from opposite monomer are marked by apostrophe (') sign. (C) Surface view of monomeric chains highlighting the residues at the dimer interface in different colors. The monomers have been separated and rotated to 90° giving clear view of interface residues. Red and blue color indicates residues involved in salt bridge formation and orange color depicts hydrophobic interactions. (D) Closer view of residues at the interface forming salt bridge. (E) Aromatic residues at the interface arranged as a stack of ring structures forming amino acids zipper. (F) Residues at the active site interacting with cofactor PLP from each monomer are presented in sticks. Residues from subunit A and B are shown in yellow and green colors respectively.

In the dimeric structure of enzyme, two active site pockets rest at the dimer interface involving the interactions of residues from both the subunits. β/α-barrel domain is the main site for cofactor PLP binding where as residues from the sheet domain of other subunit interacts with the substrate L-ornithine to form the complete catalytic pocket for enzymatic activity. The subunits associate in a head to tail manner (Figure 9). The dimeric structure is stabilized by various polar interactions present between the two subunits at the dimer interface as shown in figure 9. However, four major salt bridges K157-D338′ and D122-R277′, D338-K157′ and R277-D122′ are observed and these have been reported to play a vital role in the dimer formation of human, mouse, and *T. brucei* ODCs [22]. These interface residues are partially hydrophilic and are highly conserved in human, mouse and *Eh*ODC. Furthermore, the most prominent feature observed near C-terminal domain is presence of a stack of aromatic rings i.e. F371′/H296′/F305 and F305′/H296/F371 which is anticipated to function as an amino acid zipper. Distal amino acid residues of the zipper participate in active site pocket formation. Further, the structural analysis revealed that the close packing of dimers shields the putative N-terminal antizyme binding loop (residues 105Tyr-132Lys) as well as the C-terminal PEST like sequence because these are concealed in between the two subunits of the dimer. Thus, it is expected that the dimerization of *Eh*ODC may be responsible for protecting *Eh*ODC enzyme from proteolytic degradation.

### Mutational analysis of dimer interface residues

Molecular model of the *Eh*ODC dimer evidently shows that the conserved catalytic residues from both monomeric subunits form two equivalent active sites at the dimer interface (Figure 2, Figure 9). Consequently, it can be hypothesized that the dimeric state of *Eh*ODC enzyme is the active form. Therefore, 3D structure based site-directed mutagenesis approach was used to examine the functional role of *Eh*ODC dimerization. Conserved residues of the catalytic pocket present at the dimer interface and also the conserved residues of the dimerization interface were mutated.

**Figure 10:**
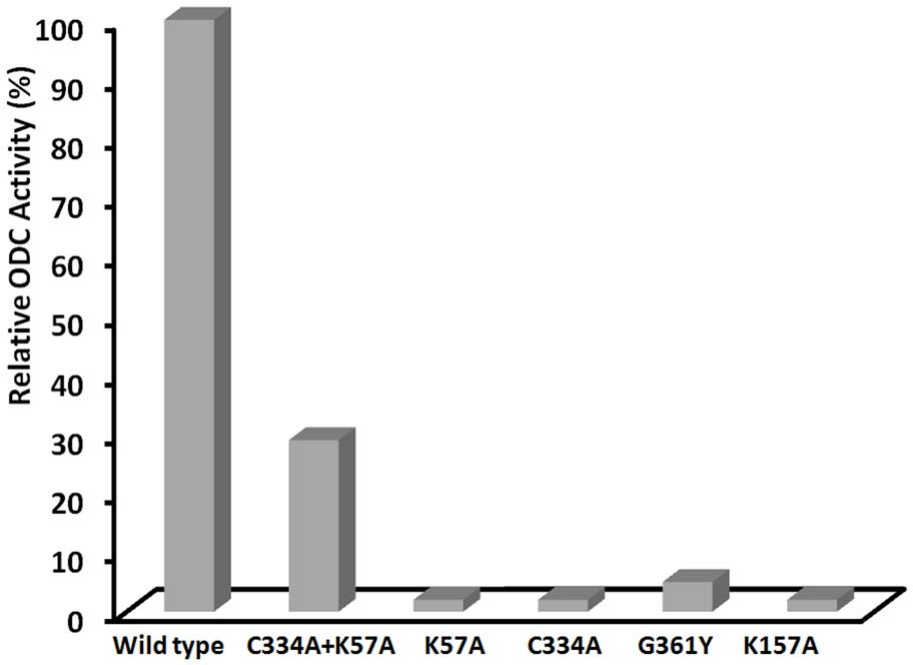
Enzyme activity of wild type *Eh*ODC and its mutants. Enzymatic activity of *Eh*ODC mutants relative to the activity of the wild-type enzyme. Cys334Ala, Lys57Ala Gly361Tyr and Lys157Ala are inactive. Cys334Ala and Lys57Ala mutants were mixed in 1∶1 ratio and the mixture shows recovery of approximately 29% of the wild-type enzyme activity. The plot represents the average of three measurements.

The conserved catalytic residues Lys57 and Cys334 present in the active site were selected for mutational studies, because the structure model of *Eh*ODC as well as the sequence alignment of *Eh*ODC with human ODC revealed that Lys57 of one subunit (Lys69 in human) and Cys334′ of other subunit (Cys360 in human) jointly play critical role in catalysis and substrate specificity in a single active site pocket (Figure 2, Figure 9) [53]–[55]. The residue Lys57 plays crucial role in PLP binding by forming Schiff base to aldehyde group with its –NH_2_ group, thus serves as a proton donor during catalysis [56]. The interaction of Lys57 with PLP governs its position and correct orientation at active site. Gel filtration analysis indicates that K57A mutant exists in the dimeric form indicating that this mutation does not disrupt dimerization (data not shown). However, when enzyme activity was examined, K57A mutation was found to abolish enzyme activity with ∼2% activity as compared to the wild type (Figure 10).

Moreover, Cys residue in the same active site from other subunit in the active site is involved in substrate binding and stabilizes the quinonoid intermediate by using its carbonyl group [54], [57]. This residue is crucial for decarboxylation of L-ornithine and release of decarboxylated product towards the interface to exit from active site. The C334A mutant was also found to be a dimer indicating that mutation does not affect dimerization (data not shown). However, C334A was also found to be inactive with ∼2% enzymatic activity as compared to wild type (Figure 10).

**Figure 11:**
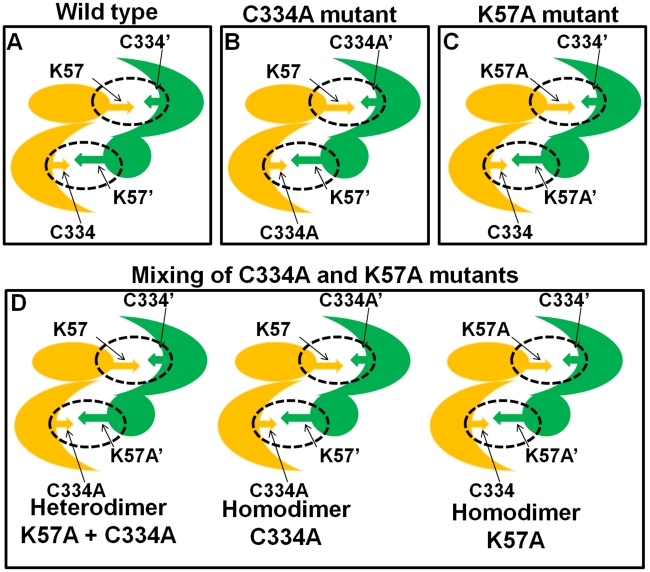
Schematic representation of homodimers and heterodimer in the mixture of *Eh*ODC Cys334Ala and Lys57Ala mutants. (A–C) Homodimer formation of wild-type and mutants of *Eh*ODC in individual solutions. (D) Possible combinations of *Eh*ODC monomeric subunits in the mixture of Cys334Ala and Lys57Ala mutants forming heterodimer and homodimers.

Interestingly, when the two mutant proteins K57A and C334A were mixed in equal concentration, the enzyme activity was partially regained having 29% activity as compared to wild type (Figure 10). The recovery of enzyme activity on mixing these two mutants is only possible when the two mutants associate to form a heterodimer. The formation of heterodimer is anticipated to restore one of the two active sites at the dimer interface as depicted in figure 11. Three types of enzyme population are expected in mutant mixture i.e. homodimers of K57A, homodimers of C334A and heterodimers of K57A and C334A. Therefore, restoration of approximately one-third of the wild-type enzyme activity in the mixture of mutants is due to the dimerization of K57A and C334A which possesses a catalytically active site pocket at one end of the heterodimer. These mutagenesis results evidently demonstrate that dimeric state is the functional form of ODC enzyme in *E. histolytica*.

**Figure 12:**
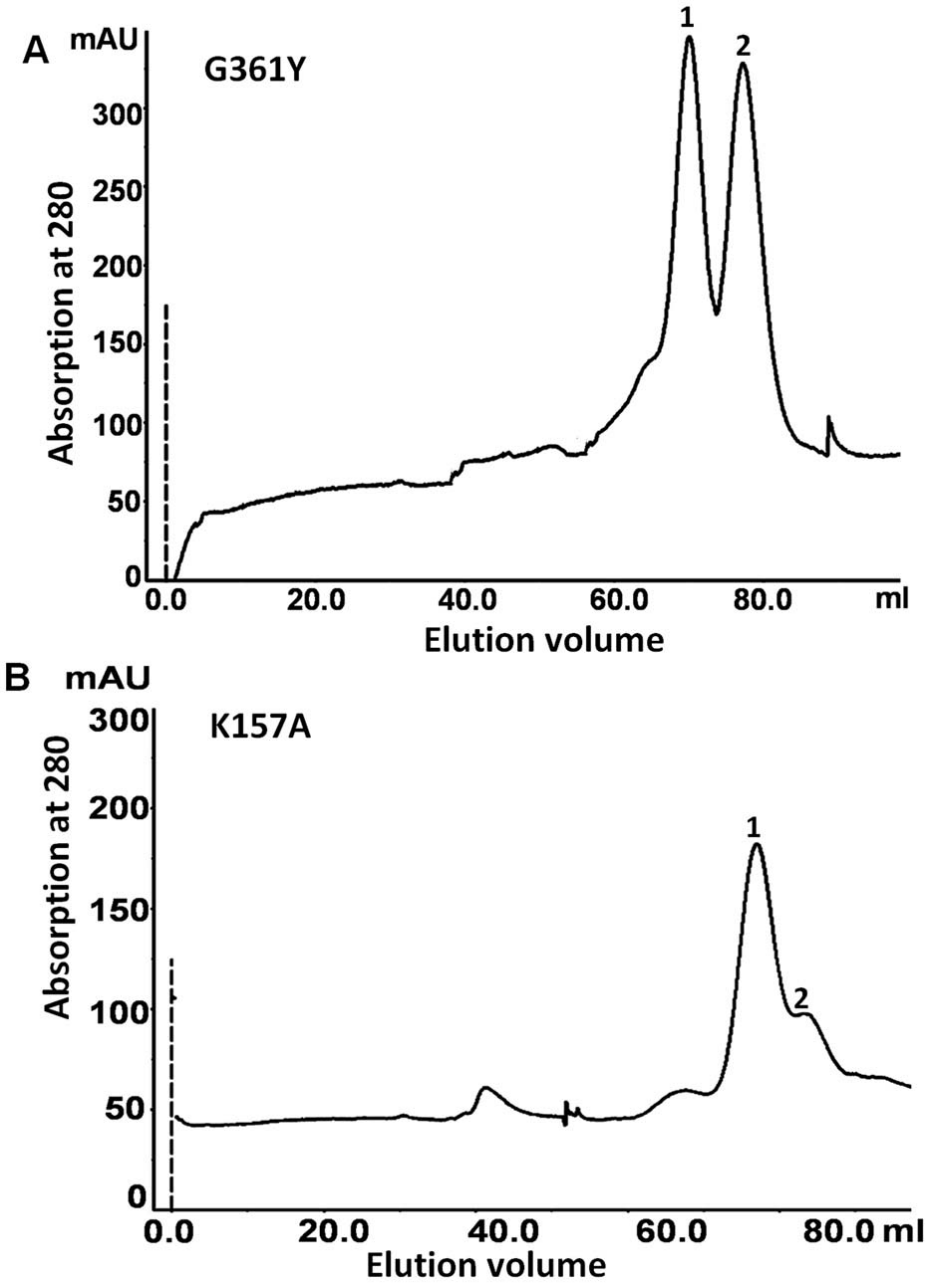
Gel filtration analysis of interface residue mutants. (A) Gel-filtration chromatogram of Gly361Tyr mutant showing partial dissociation of dimers into monomers; (B) Gel-filtration chromatogram of Lys157Ala mutant showing partial dimeric disruption.

In mouse, 19 conserved residues at the dimer interface were mutated to identify the key residues responsible for dimerization [48]. It was noted that substitution of conserved Gly387 to any amino acid except alanine abolished the enzymatic activity. The same result is also observed in case of *Lactobacillus* and hamster, where the corresponding glycine was mutated to any bulky amino acid resulted in inactivation of the enzyme [58], [59]. Crystal structure of mouse ODC revealed that this mutation could position β/α-barrel at a different angle to β-sheet so that in the mutant protein these domains have different orientations in the dimer compared to the wild type which makes the enzyme inactive [22]. In the present study, *Eh*ODC Gly361 (Gly387 in mouse) was mutated to bulky Tyr residue and its influence on dimerization was assessed by gel filtration analysis. The chromatogram showed partial destabilization of dimer with two distinct peaks corresponding to the molecular weight of monomer and dimer (Figure 12). The examination of enzyme activity showed that the Gly361Tyr mutant is functionally inactive (Figure 10). These results suggest that Gly361 in *Eh*ODC is not involved in direct interaction between the two subunits of dimer, however it plays an indirect role in the dimer stability through long range molecular interactions.

Additionally in the structure model and sequence alignment analysis, Lys157 of *Eh*ODC is conserved and forms a salt bridge with Asp338′ connecting the two monomeric subunits. At the same position in the crystal structure of human ODC, Lys169 of one subunit is involved in the salt bridge formation with Asp364′ of other subunit near the active site [21], [22]. Thus, Lys157 of *Eh*ODC plays a critical role in spatial arrangement of active site residues from both the subunits in a proper orientation along with its role in dimer formation. Mutation of Lys157 to Ala (K157A) leads to inactivation of enzyme (Figure 10). Moreover, partial disruption of the dimer as compared to the wild type protein was observed for K157A mutant, because a peak corresponding to the monomeric state of *Eh*ODC along with the dimer peak was observed in the gel filtration chromatogram (Figure 12). These results suggest that Lys157 plays a direct role in dimerization that eventually leads to the active site formation.

Furthermore, a double mutant of *Eh*ODC having two mutations i.e. G361Y and K157A was expressed in *E. coli*. The protein was over-expressed using high IPTG concentration of ∼2 M for induction. This double mutant was found to be unstable and susceptible to protease degradation during purification. Therefore, it could not be purified for further analysis. The instability of the double mutant G361Y and K157A could be due the dimer disruption making the protein insoluble as well as proteolytically unstable.

### Conclusion

Our current study, evidently demonstrates that *Eh*ODC enzyme exists in the dimeric form. The role of dimerization with respect to functionality was investigated by comparative structure modeling and mutational studies. Molecular structure reveals a sharp complementary arrangement of interface and active site residues to support the proper spatial arrangement. Thus, it contributes both the subunits in generation of two equivalent active sites. The partial recovery of the enzyme activity on mixing the two mutants, C334A and K57A which were individually inactive, shows that dimer is the active form of *Eh*ODC. Additionally, a single substitution at G361Y resulted in partial destabilization of the dimer and renders the enzyme inactive. Further, K157A mutation expected to disrupt a salt bridge K157-D338′ between two subunits didn't completely disrupt the dimer but inactivates the enzyme. These results signify that various long and short range forces play a crucial role in the dimerization and the geometry of the dimer interface is ideal for enzyme activity. Based on these observations, it can be proposed that disruption of functional *Eh*ODC dimer could be a novel target for anti-amoebiasis drugs. Molecular 3D model of *Eh*ODC dimer may support and open possibilities to find new structure based inhibitor molecules for the enzyme.
